# Changes of attachment characteristics during psychotherapy of patients with social anxiety disorder: Results from the SOPHO-Net trial

**DOI:** 10.1371/journal.pone.0192802

**Published:** 2018-03-08

**Authors:** Bernhard Strauß, Uwe Altmann, Susanne Manes, Anne Tholl, Susan Koranyi, Tobias Nolte, Manfred E. Beutel, Jörg Wiltink, Stephan Herpertz, Wolfgang Hiller, Jürgen Hoyer, Peter Joraschky, Björn Nolting, Viktoria Ritter, Ulrich Stangier, Ulrike Willutzki, Simone Salzer, Eric Leibing, Falk Leichsenring, Helmut Kirchmann

**Affiliations:** 1 Institute of Psychosocial Medicine and Psychotherapy, University Hospital, Jena, Germany; 2 Anna Freud National Centre for Children and Families, London, United Kingdom; 3 Department of Psychosomatic Medicine and Psychotherapy, University Medical Center, Johannes Gutenberg University, Mainz, Germany; 4 Psychosomatic Medicine and Psychotherapy, LWL University Clinic, Bochum, Germany; 5 Clinical Psychology and Psychotherapy, Johannes Gutenberg University, Mainz, Germany; 6 Clinical Psychology and Psychotherapy, Technical University, Dresden, Germany; 7 Department of Psychotherapy and Psychosomatic Medicine, University Hospital, Technical University, Dresden, Germany; 8 Psychosomatic Hospital, Esslingen, Germany; 9 Clinical Psychology and Psychotherapy, Johann Wolfgang von Goethe University, Frankfurt, Germany; 10 Department of Psychology and Psychotherapy, University of Witten-Herdecke, Witten-Herdecke, Germany; 11 International Psychoanalytic University, Berlin, Germany; 12 Psychosomatic Medicine and Psychotherapy, University Medicine, Georg-August-University, Göttingen, Germany; 13 Psychosomatic and Psychotherapy, University Hospital Justus-Liebig-University, Giessen, Germany; Brown University, UNITED STATES

## Abstract

**Objectives:**

Within a randomized controlled trial contrasting the outcome of manualized cognitive-behavioral (CBT) and short term psychodynamic therapy (PDT) compared to a waiting list condition (the SOPHO-Net trial), we set out to test whether self-reported attachment characteristics change during the treatments and if these changes differ between treatments.

**Research design and methods:**

495 patients from the SOPHO-Net trial (54.5% female, mean age 35.2 years) who were randomized to either CBT, PDT or waiting list (WL) completed the partner-related revised Experiences in Close Relationships Questionnaire (ECR-R) before and after treatment and at 6 and 12 months follow-up. The Liebowitz Social Anxiety Scale (LSAS) was administered at pre-treatment, post-treatment, and at 6-month and 1-year follow-up. ECR-R scores were first compared to a representative healthy sample (n = 2508) in order to demonstrate that the clinical sample differed significantly from the non-clinical sample with respect to attachment anxiety and avoidance.

**Results:**

LSAS scores correlated significantly with both ECR-R subscales. Post-therapy, patients treated with CBT revealed significant changes in attachment anxiety and avoidance whereas patients treated with PDT showed no significant changes. Changes between post-treatment and the two follow-ups were significant in both conditions, with minimal (insignificant) differences between treatments at the 12- month follow-up.

**Conclusions:**

The current study supports recent reviews of mostly naturalistic studies indicating changes in attachment as a result of psychotherapy. Although there were differences between conditions at the end of treatment, these largely disappeared during the follow-up period which is line with the other results of the SOPHO-NET trial.

**Trial registration:**

Controlled-trials.com ISRCTN53517394

## Introduction

Attachment theory postulates that early experiences play a crucial role in shaping a child’s expectations concerning the caregivers’ responsiveness and trustworthiness to their child’s attachment needs [1]. Actual dyadic experiences and related expectations are internalized into internal working models of attachment in later life, reflecting basic beliefs about oneself as worthy, loveable, and competent and about others as available and responsive. Securely attached adults consider themselves as worthy of care and affection of others, perceive significant others as trustworthy and well-intentioned, and they find it easy to get close to others [2]. They normally do not worry about being abandoned. Adults with anxious–preoccupied attachment have negative internal working models of themselves but positive ones of significant others. This can lead to relationships that are characterized by a constant worry about abandonment, a hypervigilance with regard to the love and support of others and a strong desire to be very close to others (e.g. [3]). In contrast, adults with an avoidant or dismissive attachment style tend to deny their emotional needs for closeness, find it hard to trust others thereby limiting their capacity for developing truly intimate relationships. An avoidant attachment pattern is characterized by avoidance of social interactions due to a negative view of others and a positive view of the self. Two major approaches to adult attachment have developed and determined research [4]: First, a developmental approach has mainly used the Adult Attachment Interview (AAI) and aims at measuring attachment by inferring states of mind regarding childhood experiences with relevant caregivers [5]. This approach usually leads to a categorical classification of attachment (i.e. autonomous, preoccupied/enmeshed, dismissing and additionally unresolved trauma/loss). A second approach, mainly developed within social psychology and personality research, commonly relies on self-report measures of attachment styles and related thoughts and feelings in adult relationships, usually assessing the degree of *attachment anxiety* and *avoidance* [6]. Within both traditions, there is ample empirical evidence related to the subtypes of attachment characteristics as well as their role for the formation of actual interpersonal experiences [2].

Accordingly, detailed research on attachment characteristics of adults (initially related to research on parenting) provided the basis for an increasing application of attachment-related research in psychotherapy. Attachment-related variables have increasingly been investigated in relation to psychotherapy during the last decades. There are, for instance, numerous studies relating attachment characteristics to outcome [7], to process variables such as the therapeutic or working alliance [8], or to the match between therapist and patient attachment and its relationship to process and outcome [9, 10].

The question whether attachment characteristics, usually seen as rather stable over the life span, change during psychotherapy has been answered insufficiently so far. In a recent review, Taylor, Rietzschel, Danquah and Berry [11] summarized a total of 14 studies investigating this question based upon either interview or self-report measures of attachment. Although these studies were very heterogeneous with regard to the patient populations (e.g., patients with Borderline Personality Disorders, Binge Eating Disorders, PTSD, “attachment injuries”), therapeutic approaches (e.g., psychodynamic psychotherapy, integrative CBT, interpersonal therapy, transference-focused therapy), methods to assess attachment (e.g., questionnaires or), treatment setting (e.g., inpatient, outpatient; individual, couple, group), and the study quality, it seems that attachment security at least slightly increases during psychotherapy. The authors consequently request to further confirm this conclusion empirically calling for more rigorous study designs including *controlled trials* comparing different treatments and their differential impact on attachment characteristics.

Using attachment theory, Nolte, Guiney, Fonagy, Mayes, and Luyten [12] have recently proposed that a predominantly anxious attachment style may predispose for anxiety disorders. Vertue [13] suggested that avoidant individuals would expect devaluation in social interaction and would be more prone to social anxiety disorder (SAD). SAD is a common psychological disorder with an early onset resulting in severe psychosocial impairment and high socioeconomic costs [14]. Comorbidity with other anxiety disorders, depression or substance abuse is common [15]. Recent epidemiological studies reveal high prevalence rates for SAD among adults (life time: 13% [16]; twelve months prevalence: 3–12%; [17–18]). According to Leary [19], attachment theory can explain “a. the motivation of social phobic patients to make a positive impression on others, b. the believe that one will fail to do so and c. the fear of resulting relational devaluation and the failure to achieve interpersonal goals”(cited by [13] p. 185). A negative internal working model of oneself and others can lead to (i.) a strong need for approval as a result of lack of or insensitive responses from primary caregivers, (ii.) the believe that there are deficits in social skills, specifically in the ability to influence others’ emotions in desired ways, and (iii.) the fear of the threat of social exclusion and rejection [19]. Several studies suggested that attachment characteristics play an important role in the development of SAD and showed, for instance, that affected patients have a higher percentage of insecure attachment compared to healthy controls (e.g. [12, 20–22]). Eng et al. [21] reported that patients with social phobia have higher levels of attachment-related anxiety and a lower degree of attachment security than a control group. Furthermore, an anxious-preoccupied attachment style was accompanied by higher levels of impairment on several symptom scales in comparison to a subsample of social phobic patients with a secure attachment style. Likewise, a secure attachment style is associated with a lower degree of social anxiety symptoms as recently shown by our own group in a clinical sample [23] and by van Buren & Cooley [24] in a non-clinical sample. Mickelson, Kessler, & Shaver [22] reported that SAD was negatively related to secure attachment and positively associated with ambivalent and avoidant attachment. For other anxiety disorders, insecure-ambivalent attachment styles were also found to be dominant [25–28]. Therefore, the consideration of attachment characteristics seems important for a comprehensive understanding of the disorder and its treatment.

The SOPHO-NET trial, a randomized controlled study comparing a waiting list condition (WL) with a short term cognitive behavioural (CBT, [29]) and a short term psychodynamic treatment (PDT, [30]) for social anxiety disorders (SAD) offered a unique opportunity to further study potential differential treatment effects in attachment-related aspects. In a large sample of N = 495 patients, a self-report measure of attachment (the ECR-R, [31]) was consistently administered across 5 time points of measurement (for details of the trial and its main results see [32–34]). The main findings indicated that both, CBT and PDT, were efficacious compared to a waiting list with respect to pre-post symptom improvement (expert ratings and self-ratings of social anxiety symptoms) and to secondary outcome measures (interpersonal problems and depression). CBT was significantly superior to PDT showing small between-group effect sizes concerning dimensional symptom improvement, remission rates, and interpersonal problems but not concerning response rates and depression [33]. In another report by Leichsenring, Salzer, Beutel et al. [34] results related to long-term outcome (2 year follow-up) were described, indicating response rates of approximately 70%, and remission rates of 40% in both treatment conditions.

Within the controlled trial a variety of secondary outcome measures and potential predictors of outcome were assessed (e.g. interpersonal problems, depressive symptoms). Among these, attachment characteristics were measured in the entire sample at the beginning and the end of psychotherapy including follow-up measures. So far, these attachment measures were only analyzed as predictors of change within both treatment arms [35, 36] as well as a moderator of change [37]. Changes of attachment characteristics, the focus of this paper, have not been analyzed within the trial so far.

## Research questions

The following questions will be addressed in this report: (1) Do patients with SAD differ from a large healthy (representative) control group with respect to attachment and are indicators of attachment insecurity (anxiety, avoidance) correlated with social anxiety symptoms? It was hypothesized that SAD patients should have higher scores, both, in the attachment anxiety as well as the avoidance scale of a self-report measure and that symptoms are correlated with these indicators. (2) Do attachment anxiety and avoidance change within the two treatment conditions compared to a waiting list condition? In accordance with the available reviews, we expected a general reduction of attachment related anxiety as well as avoidance in both treatment conditions. (3) Are there interaction effects indicating that attachment characteristics respond differentially to the two treatment formats. Although the previous results of the trial would indicate no differences between the treatments, it was assumed that, due to its interpersonal focus, PDT might have stronger effects on self-rated attachment characteristics.

## Materials and methods

### The SOPHO-NET study

As mentioned above, the SOPHO-Net trial including its design and implementation has been extensively described elsewhere [33]. The major results related to the primary and secondary outcome criteria were reported in two major publications [34, 35]. The study protocol was approved by the ethics committee of the Georg -August-University Göttingen, where the multisite study was coordinated. The clinical trial was monitored by the Coordination Center for Clinical Trials at the University of Heidelberg, which was independent of the participating research centers.

Both manual-guided treatments comprised up to 25 individual (50-minute) treatment sessions. The mean (±SD) number of completed sessions was 25.84±9.13 for CBT and 25.67±9.61 for psychodynamic therapy; the mean duration of treatment was 38.69±16.03 weeks for CBT and 37.40±18.03 for PDT (p = .51 [33]). Patients included in this study had to be 18–70 years of age and to have a diagnosis of social anxiety disorder according to the Structured Clinical Interview for DSM-IV (SCID) [38] (German version), to have a score >30 on the Liebowitz Social Anxiety Scale [39], and to have a primary diagnosis of social anxiety disorder according to the rating on the Anxiety Disorders Interview Schedule [40]. To increase the external validity of the trial patients with all comorbid mental disorders less severe than social anxiety disorder (according to the Anxiety Disorders Interview Schedule rating) were included except those exhibiting the following exclusion criteria: psychotic and acute substance-related disorders; cluster A and B personality disorders; prominent risk of self-harm; organic mental disorders; severe medical conditions; and concurrent psychotherapeutic or psychopharmacological treatments (for details see [33, 34]).

### Measures

Within the SOPHO-Net trial a battery of measures related to symptom load and other psychological characteristics were used including clinical interviews to confirm the inclusion and exclusion criteria.

To analyse the above-mentioned questions, we used the following measures: *Experiences in Close Relationships Questionnaire (ECR-Revised*, *German Version)*. The original questionnaire was revised by Fraley et al. [31] to promote consensus on the most appropriate method for assessing self-reported adult romantic attachment. The 33 item 7 point Likert scale German version was translated and validated by Ehrenthal, Dinger, Lamla, Funken, and Schauenburg [41]. The ECR-R consists of two scales/dimensions, with attachment-related anxiety reflecting insecurity about the availability and responsiveness of romantic partners and the attachment related avoidance dimension corresponding to feeling uncomfortable about being close or depending on intimate others. In the current study, Cronbach’s alpha was .93 for the anxiety scale and .92 for the avoidance scale. In the healthy control sample from a representative data survey, a 12 item short version of the ECR-R was used reflecting a similar factor structure [42]. For the comparison of the two samples subscales were selected from the 33-item instrument relfecting the 12 items of the short form.

*Liebowitz Social Anxiety Scale (LSAS)*. SAD was assessed through the German version of the LSAS [39, 43] by trained clinicians. On 4 point scales, the 24 item questionnaire assesses on separate scales the amount of anxiety in social and performance situations (e.g. meeting strangers, giving a talk) and the frequency of avoiding these situations. As for the original version, a high interrater reliability (r = .84), the ability to classify individuals with and without SAD and internal consistency has been demonstrated for the German version. The LSAS total scores showed good internal consistency in a German study (α = .94) [43]

### Sample

Patients were recruited by outpatient clinics at the universities of Bochum, Dresden, Göttingen, Jena and Mainz (Germany). The initial sample of the trial consisted of 1450 patients screened within the five study centres for inclusion criteria, 514 –after informed consent—were initially randomized and 495 randomly assigned to the treatment and waiting list conditions (209 CBT, 207 PDT and 79 WL). 159 patients completed the CBT and 149 the PDT [33]. WL patients were also randomized to one of the active treatment conditions after the end of the waiting period. Post-therapy assessments were available from 159 patients in the CBT condition per protocol, and 149 patients in the psychodynamic treatment condition. We used the initial samples of each treatment condition with valid scores in the ECR (i.e. a total of 474 patients, 244 in the CBT and 230 in the PDT condition). The average age was 35.2 years (*SD* = 12.2), percentage of females was 54.7% (cf. Table 1).

**Table 1:** Characteristics of the samples (Representative sample, SOPHO-Net sample), CBT, PDT = treatment subsamples.

	Rep. Sample (N = 2508)		SOPHO-Net (N = 474)		CBT (N = 244)		PDT (N = 230)	
	M	SD	M	SD	M	SD	M	SD
Age	49,7	(18,3)	35,1	(9,1)	35,3	(11,8)	34,9	(12,2)
% Females	53,2%		55,0%		57,0%		52,8%	
ECR anxiety (short version)	2,4	(1,3)	3,7	(1,1)	3,7	(1,5)	3,8	(1,5)
ECR avoidance (short version)	2,6	(1,4)	2,8	(0,9)	2,8	(1,2)	2,8	(1,3)
ECR anxiety (long version)	-	-	3,5	(1,2)	3,5	(1,3)	3,6	(1,3)
ECR avoidance (long version)	-	-	3,0	(1,1)	3,0	(1,1)	3,0	(1,2)
LSAS anxiety	-	-	39,7	(11,0)	39,3	(11,2)	40,0	(10,9)
LSAS avoidance	-	-	33,2	(12,0)	32,8	(12,1)	33,6	(11,9)

To test the first hypothesis, we used a representative (non-clinical) sample of 2508 German citizens who completed a short (12 item) version of the German ECR questionnaire as part of a larger representative survey collected a variety of psychometric data for several research groups such as, for example, the prevalence of self-harm or somatic complaints [44]. This representative sample consisted of 53.2% females and 46.8% males with a mean age of 49. 7 years. The ECR was part of a battery of questionnaires used within a nation-wide representative face-to-face household survey conducted in Germany. The survey was carried out by professional interviewers of a demographic consultation company (USUMA, Berlin) in the year 2015. The survey met the ethical guidelines of the international code of marketing and social research practice by the International Chamber of Commerce and the European Society of Opinion and Marketing Research. A representative sample of the general German population aged 14 years or older was approached using 258 sample points (cf. Table 1).

### Statistical analysis

To test the first hypothesis, we analysed the influence of the group (clinical vs. non-clinical), age as well as sex on the dependent variables (ECR-R anxiety and avoidance scales). This was done using a generalized analysis of covariance (g-ANCOVA, cf. [45]). We then calculated adjusted means for both samples to control for non-randomisation. Finally, the adjusted means were compared using a t-test for independent samples. For all descriptive statistics and Spearman correlations to determine the relationship between social anxiety symptoms and attachment anxiety/avoidance we used IBM SPSS Statistics 20. Comparisons of the clinical and non-clinical sample were performed using the program M-Plus.

Changes in attachment anxiety and avoidance were analysed using generalised analysis of covariance based upon the software EffectLite v.3.1.3 using a multi-sample structural equation approach in order to compute treatment effects (for details cf. [46]). g-ANCOVA has the advantage to allow variations of treatment effects depending on the covariates (i.e.initial ECR- and LSAS-scales). It does not require homogeneity of variance, and it uses full information maximum likelihood estimation (FIML). FIML is superior to listwise or pairwise deletion if data missing completely at random is not given [47]. In addition, effect sizes were calculated (Cohen´s d).

To test the questions if attachment anxiety and avoidance change during the treatments and if these changes interact with the treatment type (CBT vs. PDT), we performed analyses of covariance using the pre-treatment scores as covariate.

The comparison of the two treatments was done based on the adjusted means estimated to test the second hypothesis. Here, the ECR means were compared for the post-treatment and follow-up measures using a t-test for independent samples (with alpha adjusted for multiple comparisons).

## Results

The SOPHO-NET sample differed significantly from the non-clinical sample both with respect to the anxiety scale of the ECR-R as well as the avoidance scale (cf. Table 2). As the effect sizes indicate, differences are considerably larger for the anxiety as compared to the avoidance scale.

**Table 2:** Comparison of the SOPHO-Net patients with a representative German sample using adjusted scale means (related to a mean age of 35.1 years and 55% females).

	R21 (N = 2508)		SOPHO-NET (N = 474)		difference					
	M	SD	M	SD	diff	SE	Z	p	SD_pooled	Cohens d
ECR-An	2,55	(1,75)	3,71	(1,48)	1,16	(0,08)	15,29	<0,0001	1,71	0,68
ECR-Av	2,59	(1,60)	2,77	(1,24)	0,18	(0,07)	2,78	0,005	1,55	0,12

ECR-An = Anxiety scale, ECR-Av = Avoidance scale

In the SOPHO-NET sample, Spearman correlations (Rho) between the ECR-R attachment scales and social anxiety (LSAS total score) indicate a slightly positive relationship between indicators of insecure attachment and symptoms of social anxiety (attachment anxiety: *r* = .29, *p* < .05 attachment avoidance: *r* = .28, *p* < .05).

To test for changes in attachment characteristics within the (initially) three groups of the SOPHO-NET sample (n = 495), we calculated effect sizes reflecting contrasts of CBT vs. WL, PDT vs. WL, and CBT vs. PDT groups with respect to pre-post changes. The two active treatment conditions were further contrasted in relation to the follow-up measures of attachment (6 months, 1 year).

**Fig 1:**
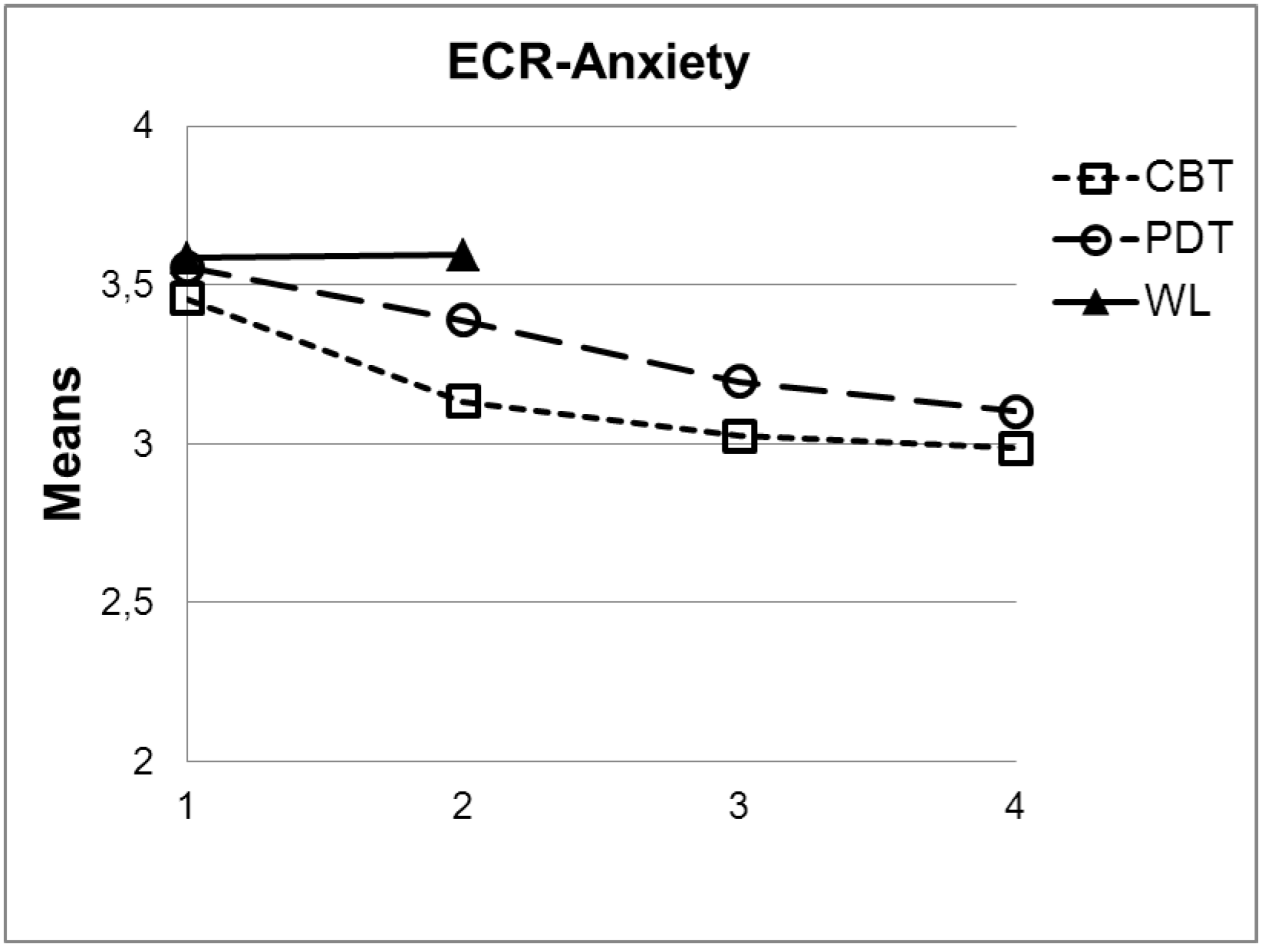
Changes of attachment anxiety (mean ECR-R score) from pre-treatment (1) to post-treatment (2) and follow-up assessments (3: 6-months, 4: 12-months) contrasting waitlist (WL, two measures only), CBT and PDT.

**Fig 2:**
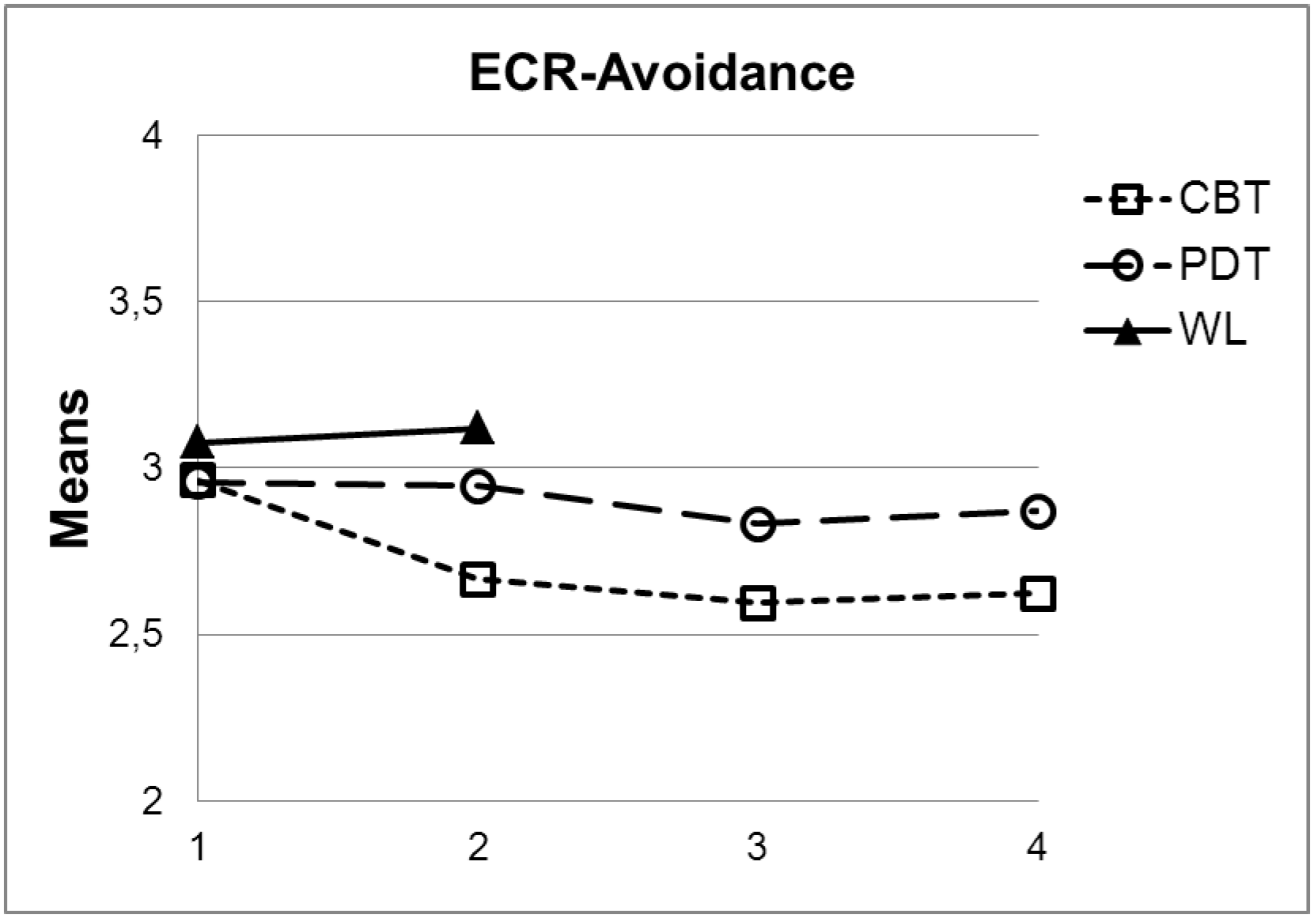
Changes of attachment avoidance (mean ECR-R score) from pre-treatment (1) to post-treatment (2) and follow-up assessments (3: 6-months, 4: 12-months) contrasting waitlist (WL, two measures only), CBT and PDT.

Figs 1 and 2 reflect the means of the two ECR subscales for the two treatment groups and the waiting list group contrasting the pre- (1), post- (2) and the two follow-up measures (6-months, 3; 12-months, 4).

Table 3 also reflects results regarding differential treatment effects and treatment type x pre-treatment attachment interactions for both scales of the ECR-R. Figs 1 and 2 show the means of the ECR-R scales.

**Table 3:** Effect sizes related to changes of attachment anxiety and avoidance comparing the different conditions.

Comparison	Average treatment effect (group difference)	
	*ES*	*p-value*
*Attachment anxiety*		
Pre-post		
CBT vs. WL	-.30	.005
PDT vs. WL	-.12	.254
CBT vs. PDT	-.16	.060
6 months follow-up		
CBT vs. PDT	-.05	.610
12 months follow-up		
CBT vs.PDT	-.11	.312
*Attachment avoidance*		
Pre-post		
CBT vs. WL	-.23	.034
PDT vs. WL	-.01	.976
CBT vs. PDT	-.24	.009
6 months follow-up		
CBT vs. PDT	-.17	.110
12 months follow-up		
CBT vs.PDT	-.19	.107

CBT = cognitive behavioral therapy, PDT = psychodynamic therapy, WL = waiting list

ES = Effect size

Within WL, the ECR-R scales did not change from pre to post (anxiety: *M*_pre_ = 3.58, *SD*_pre_ = 1.14 vs. *M*_post_ = 3.59, *SD*_post_ = 1.16; *t*(53) = .29, *p* = .77; avoidance: *M*_pre_ = 3.08, *SD*_pre_ = 1.04 vs. *M*_post_ = 3.12, *SD*_post_ = 1.12; *t*(53) = -.03, *p* = .987). As can be seen in Table 3, compared to WL, CBT patients showed significant pre-post improvement with regard to both attachment anxiety (*ES* = -.30) and avoidance (*ES* = -.23). In contrast, PDT patients did not improve significantly compared to WL. Accordingly, pre-post comparison between CBT and PDT showed better improvement for CBT patients, although—with respect to attachment anxiety—the contrast was not statistically significant using two-tailed tests (*p* = .60). With regard to long-term treatment effects we did not find statistically significant differences between the treatment groups. Moreover, no significant treatment type x pre-treatment attachment interactions could be detected.

As Fig 1 shows, attachment anxiety scores further decreased during the follow up period. In both treatment conditions, changes from post-treatment to 12-months follow-up were statistically significant (CBT: *M*_post_ = 3.13, *SD*_pre_ = 1.21 vs. *M*_12-months_ = 2.99, *SD*_12-months_ = 1.27; *t*(97) = 2.21, *p* = .03; PDT: *M*_post_ = 3.39, *SD*_pre_ = 1.23 vs. *M*_12-months_ = 3.10, *SD*_12-months_ = 1.22; *t*(89) = 2.45, *p* = .02). In contrast, attachment avoidance remained rather stable after treatment with no differential treatment effects (CBT: *M*_post_ = 2.67, *SD*_pre_ = .99 vs. *M*_12-months_ = 2.62, *SD*_12-months_ = 1.03; *t*(97) = -.23, *p* = .82; PDT: *M*_post_ = 2.95, *SD*_pre_ = 1.07 vs. *M*_12-months_ = 2.87, *SD*_12-months_ = 1.09; *t*(88) = .93, *p* = .36).

## Discussion and conclusions

This report focuses primarily on changes in patients’ attachment style measured with the Experiences in Close Relationship Scale (ECR-D), currently the most widely used self-report measure to assess attachment. Similar to other studies, we could confirm that patients suffering from SAD seem to be characterized by more insecure attachment characterized by both, higher attachment anxiety and avoidance scores than healthy controls [20–23] and that higher symptom load as measured with the LSAS was positively associated with attachment anxiety and avoidance. This results supports the view that insecure attachment might be an important factor in the development of SAD [12, 19]. The fact that both, attachment anxiety and avoidance are positively correlated with social anxiety is in line with the exting theories of anxious attachment being a general risk factor for anxiety disorders [12] and avoidance being specifically important for the development of social anxiety [13]. Interestingly, the clinical sample did not differ largely from the healthy sample in the avoidance scale which underlines Nolte et al.´s notion of the specific relevance of attachment anxiety for developing SAD [13].

A differential analysis of changes within the attachment scales during treatment revealed a) no significant changes during a waiting list period, and b) significant pre-post decreases of attachment anxiety (ES = -.30) and avoidance (ES = -.23) in the CBT condition. The changes were rather stable over the follow-up period. One limitation of the study is the fact that there is a lack of longer-term follow up data in control group.

On the other hand, differences between the treatment conditions were not or only marginally significant at 6-months and 12 months follow-up. This result is in line with the general findings provided by this trial, indicating slightly higher effects for the CBT condition if pre-post measures are concerned [34, 35], although it was expected that PDT would be superior with repect to interpersonal changes. Rapid change of symptoms might also initiate interpersonal changes in SAD, especially since the symptoms are closely related to interpersonal functioning (e.g. social withdrawal, avoidance behaviour). In addition, it can be assumed that changes in social skills—which are addressed in CBT—could also improve the inner working model related to attachment. Obviously, patients characterized by very high pre-treatment attachment avoidance have shown to specifically benefit from CBT than from PDT as was recently demonstrated in a moderator analysis of the SOPHO-NET data [37]. This might explain the immediate effect of CBT on self-reported avoidance.

With regard to attachment anxiety further improvement after treatment could be observed in both treatment conditions whereas attachment avoidance remained stable in the period after treatment. Since this pattern of self-reported change in attachment anxiety vs. avoidance stability has been reported in other studies (e.g [48, 49]), there is now converging evidence that it is easier for social anxiety patients to develop an increased feeling of self-confidence (not fearing to be disliked and abandonment by others) after treatment than it is to overcome feeling uncomfortable if others come closer, or depending on intimate others. Focusing on these issues could be an important task both, within PDT and CBT approaches to social anxiety disorders. It will be particularly interesting to shed further light onto which other psychotherapeutic mechanisms of change, including process variables such as the helping alliance, might parallel or precede attachment-related changes within the two treatment conditions.

Regarding the more general question whether and how attachment-related characteristics change during psychotherapy, our results—based upon a rigorous RCT design—confirm the conclusion of the recent review by Taylor et al. [11]. Therein the authors observed a slight increase in indicators of attachment security across several measures, treatments, disorders, and settings. In our study, the ECR-R, recently confirmed as a reliable measure of attachment [50], was used. In the review by Taylor et al. [11], three of 14 studies also measured attachment characteristics using the ECR-R drawing similar conclusions regarding a decrease of anxiety and avoidance during psychotherapy [51–53]. This could be interpreted as a general effect of any treatments, independent of the psychological disorder or the specific treatment approach. Nevertheless, our study opens up further discussion about differential treatment effects related to patient attachment, about the mechanisms of change underpinning these effects and how these mechanisms can be reproduced using specific research designs.
